# Lymphoepithelial cyst mimicking pancreatic cancer: a case report and literature review

**DOI:** 10.1186/s40792-021-01191-x

**Published:** 2021-04-29

**Authors:** Takuya Iguchi, Akira Shimizu, Koji Kubota, Tsuyoshi Notake, Shinsuke Sugenoya, Kiyotaka Hosoda, Koya Yasukawa, Hikaru Hayashi, Ryoichiro Kobayashi, Yuji Soejima

**Affiliations:** Division of Gastroenterological, Hepato-Biliary-Pancreatic, Transplantation and Pediatric Surgery, Department of Surgery, Shinshu University School of Medicine, 3-1-1 Asahi, Matsumoto, Nagano 390-8621 Japan

**Keywords:** Lymphoepithelial cyst, FDG-PET, Pancreatic cancer

## Abstract

**Background:**

Pancreatic lymphoepithelial cyst (LEC) is a rare nonmalignant cyst consisting of a benign collection of keratinizing squamous epithelial cells with lymphoid tissue. Diagnosing LEC preoperatively is considered difficult because of its non-specific clinical features; therefore, LEC is generally treated the same as a malignant tumor.

**Case presentation:**

Our case was a 65-year-old man who underwent pancreatoduodenectomy 3 years previously for carcinoma arising from the ampulla of Vater. A pancreatic mass in the remnant pancreatic tail was detected through follow-up abdominal contrast-enhanced computed tomography (CT). This revealed two adjacent ring-enhanced masses that had been in tight contact with the left diaphragm and were enlarged. The tumors had high signal intensity in diffusion-weighted images of magnetic resonance imaging, and fluorodeoxyglucose-positron emission tomography (FDG-PET) showed abnormal uptake (standardized uptake value maximum: 17.4). Therefore, we conducted a partial resection of the remnant pancreas with concomitant resection of the left diaphragm. Microscopically, one of the tumors revealed rare fragments of apparently benign squamous epithelium on a background of keratinous debris, cyst contents, and scattered lymphocytes, and the adjacent mass revealed infiltrated neutrophils. The histopathological diagnosis was an LEC with chronic abscess. The patient recovered uneventfully and was discharged on postoperative day 10.

**Conclusions:**

We reported a rare case of LEC with chronic abscess that was positively visualized on FDG-PET. When a pancreatic malignancy cannot be excluded, surgical resection is considered inevitable.

## Background

Pancreatic lymphoepithelial cyst (LEC) is a relatively rare benign cyst consisting of a collection of keratinizing squamous epithelial cells with lymphoid tissue [1]. Accurate preoperative diagnosis is of considerable clinical importance to avoid unnecessary surgery. Endoscopic ultrasound guided fine-needle biopsy (EUS-FNA) is reported to be a useful modality for detecting the pathological features of LEC [2]. However, biopsy for a potential malignant tumor cannot be easily applied from the perspective of dissemination [3]. Therefore, it is important to make the diagnosis non-invasively if possible. However, this is often difficult because LEC does not have specific clinical features, including those that can be detected by computed tomography (CT) or magnetic resonance imaging (MRI). Recently, several researchers [4, 5] reported the efficacy of fluorodeoxyglucose-positron emission tomography (FDG-PET) in diagnosing LEC. We herein present a case of LEC with chronic abscess in which abnormal uptake was obtained on FDG-PET.

## Case presentation

A 65-year-old man was referred to our hospital for follow-up of gastric cancer and carcinoma of the ampulla of Vater. In the former, total gastrectomy with splenectomy was performed 3 years previously. In the latter, pancreaticoduodenectomy was performed 16 years previously. Follow-up contrast-enhanced CT revealed a cystic lesion in the remnant pancreatic tail. Laboratory findings showed that serum levels of carcinoembryonic antigen, carbohydrate antigen 19-9 (CA19-9), elastase-1, span-1, and dupan-2 were within normal limits, as were serum levels of amylase and lipase. White blood cells, including neutrophils, lymphocytes, and platelets, were also within normal limits. He had no medical history or experience of pancreatitis, postoperative pancreatic fistula, or abdominal abscess. There was no sign of blood examination or symptom suggesting inflammation during follow-up.

**Fig. 1:**
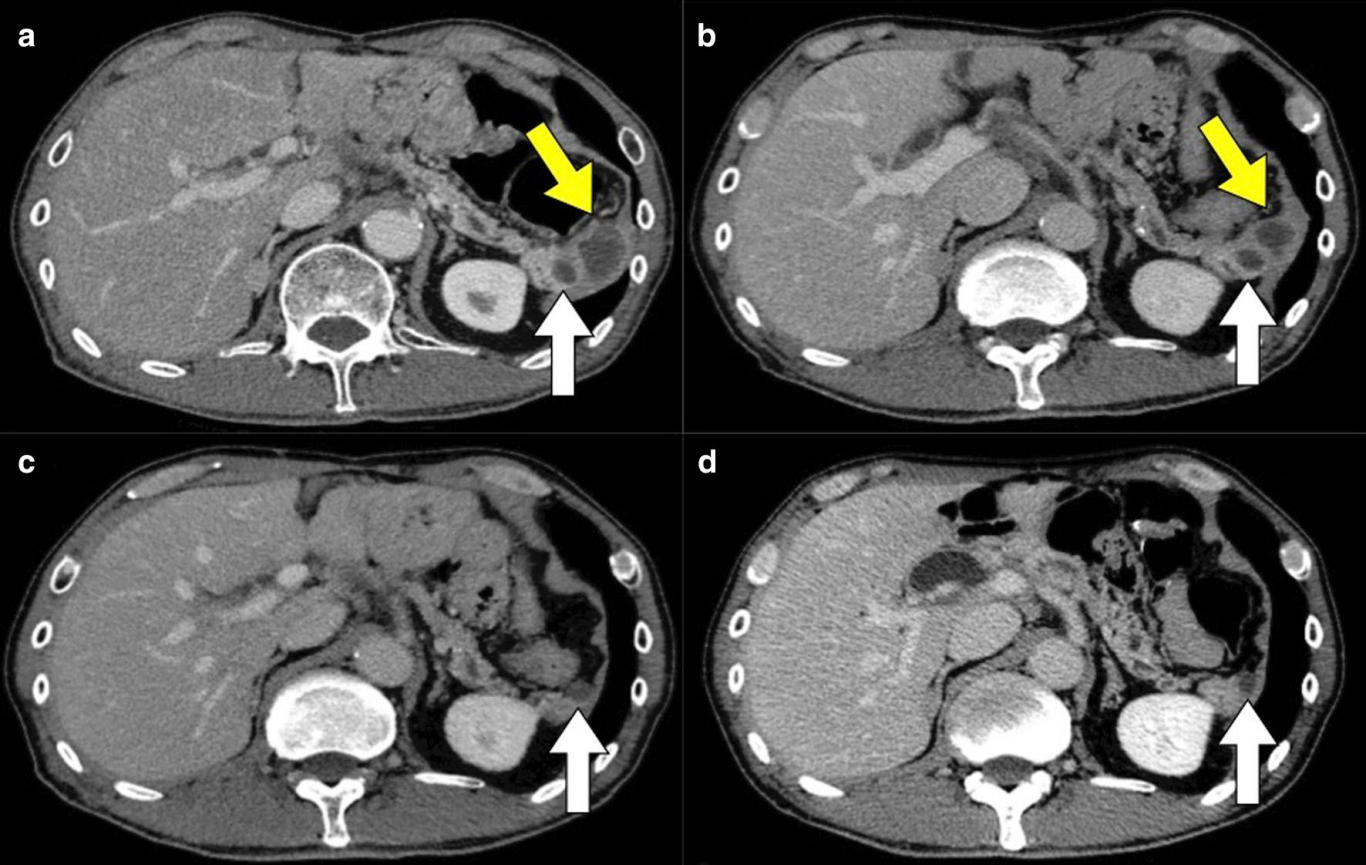
Contrast-enhanced computed tomography (CT) findings. CT showed a relatively clear, round, ring-enhanced tumor of 15 mm in diameter (white arrow) and an adjacent tumor of 35 mm in size (yellow arrow) (**a**). The larger tumor was enlarged compared with 1 month before (**b**) and had not been recognized 6 months previously (**c**). The smaller tumor had not changed from 3 years previously (**d**)

**Fig. 2:**
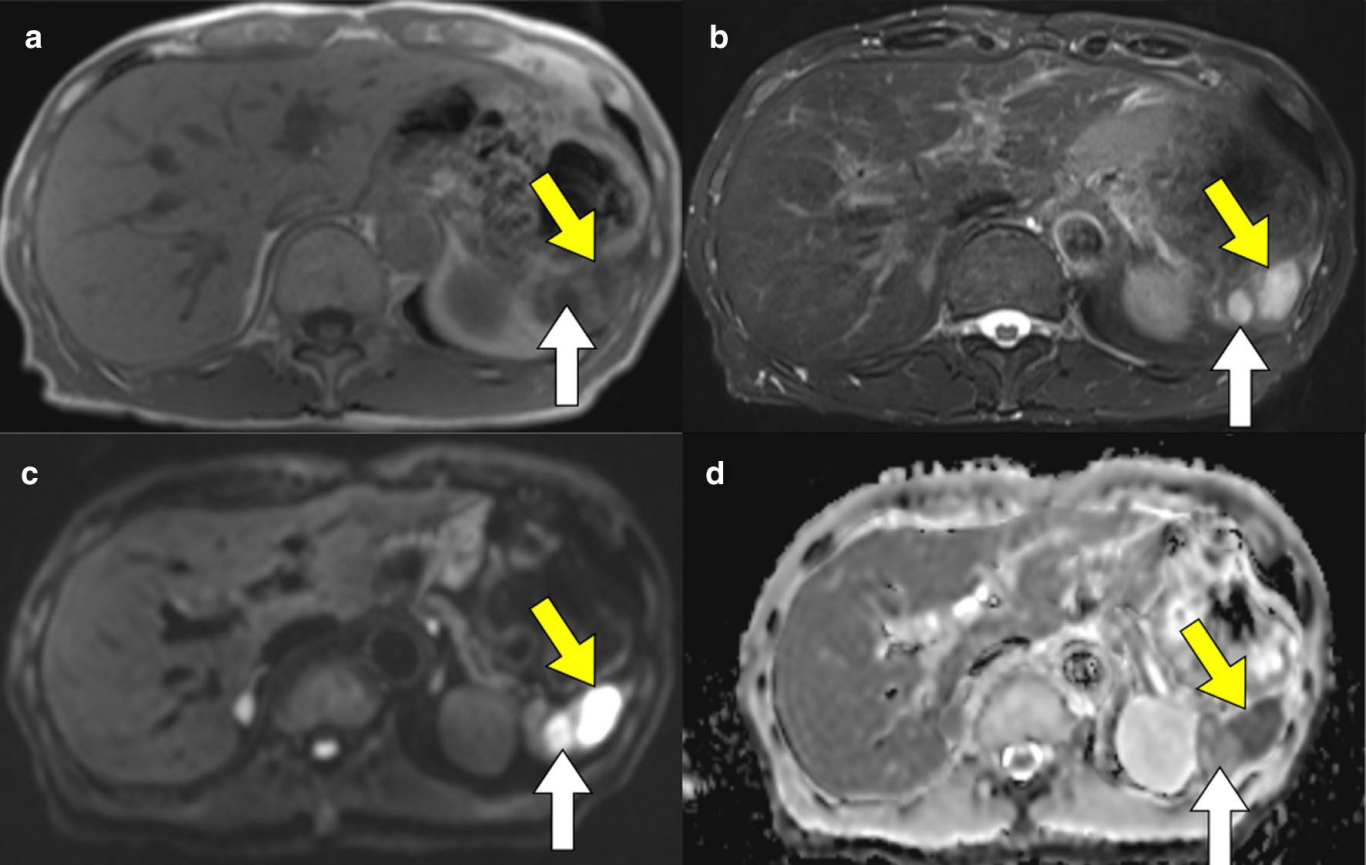
Magnetic resonance imaging findings. Magnetic resonance imaging revealed both the larger (yellow arrow) and smaller (white arrow) tumors as having low signal intensity on T1-weighted MRI (**a**), heterogeneous high intensity on T2-weighted MRI (**b**), and high signal intensity in diffusion-weighted imaging (**c**), and the apparent diffusion coefficient value revealed low signal intensity (**d**)

**Fig. 3:**
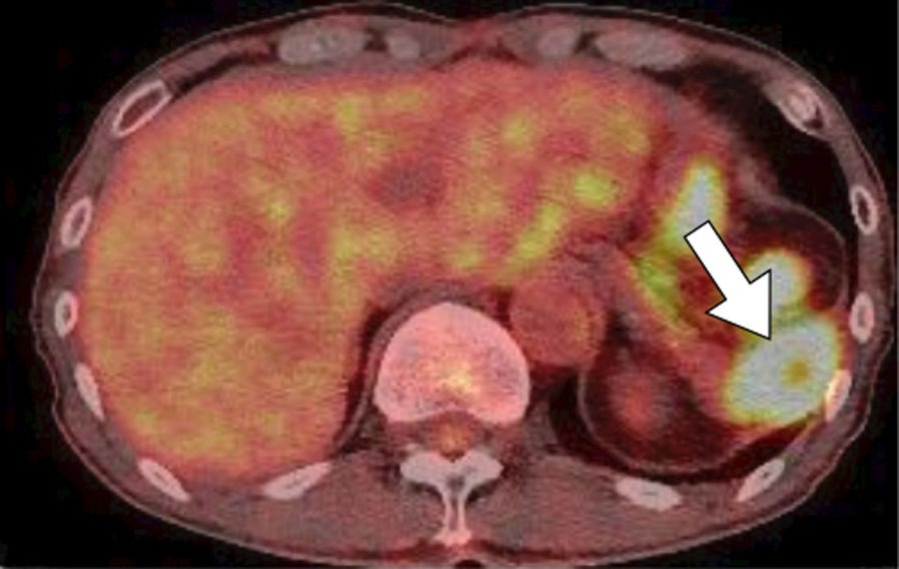
Fluorodeoxyglucose-positron emission tomography findings. The tumors were visualized as masses with a standardized uptake value maximum of 17.4 (white arrow)

**Fig. 4:**
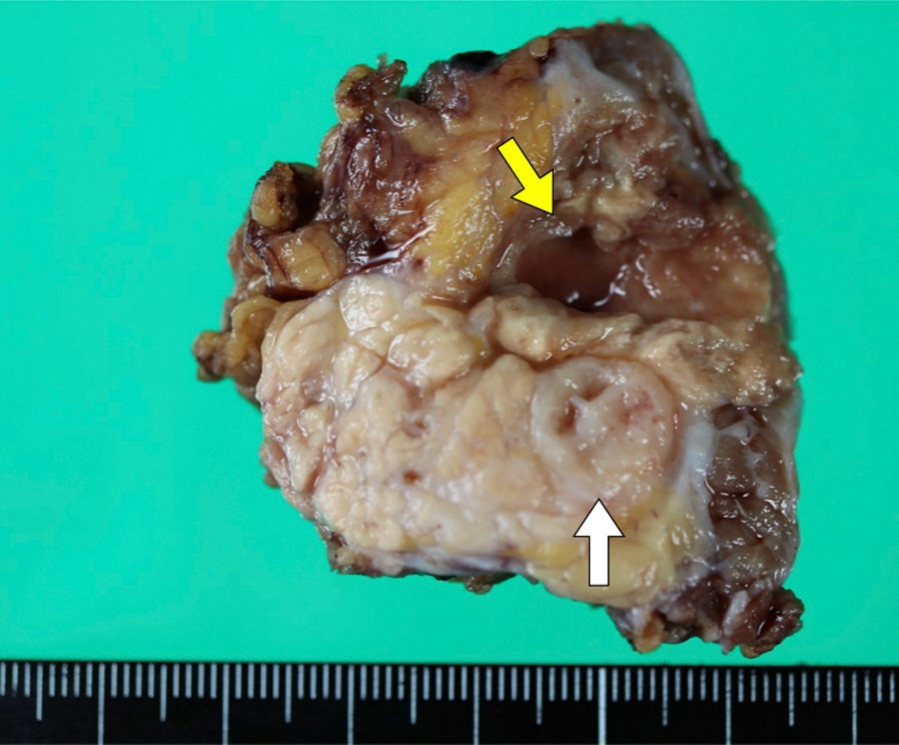
Cross sections of the resected specimen. The cystic lesion of 23 mm in diameter was filled with white-yellowish material in the pancreatic tissue (yellow arrow). The other white solid mass of 9 mm in diameter was present with intervening pancreatic tissue (white arrow)

**Fig. 5:**
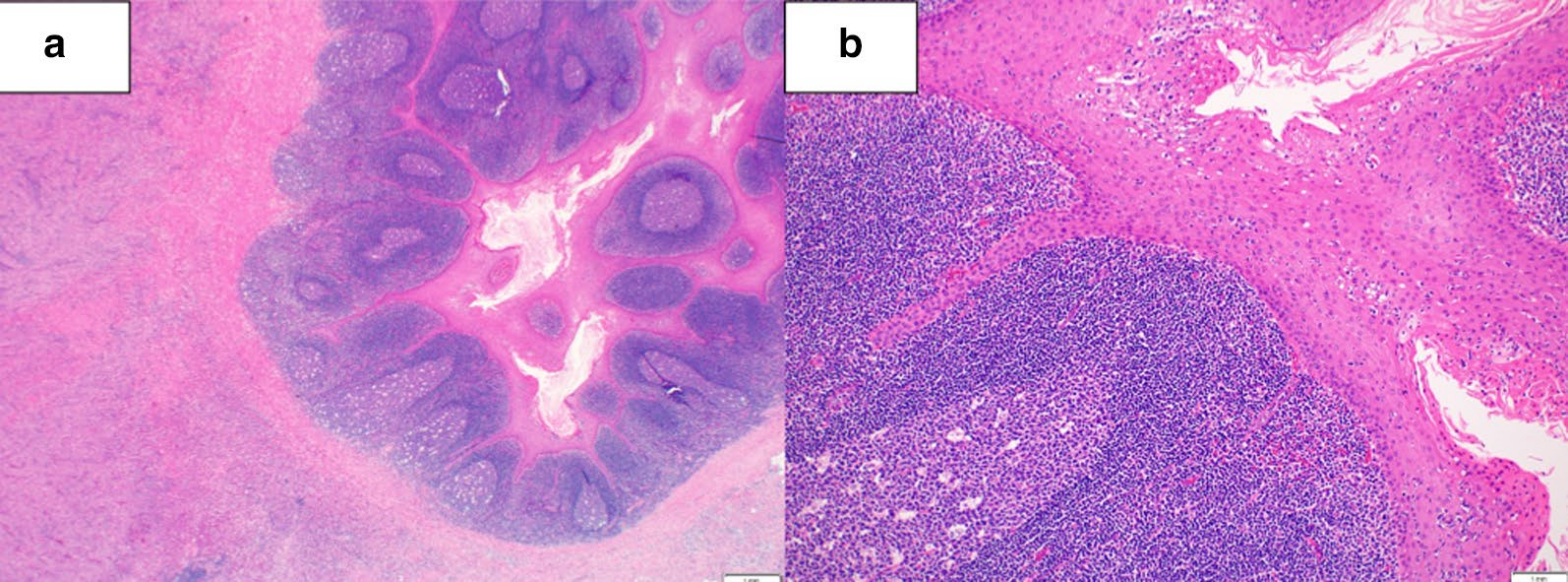
Pathological findings. Squamous epithelium on a background of cysts with proliferated lymphoid tissue was identified (**a**). Infiltration of neutrophils into the stratified squamous epithelium was evident (**b**) [2]

Abdominal contrast-enhanced CT revealed a mass of 36 mm in diameter at the tail of the remnant pancreas, with an adjacent mass of 15 mm (Fig. 1a). These two masses were almost clear and round and appeared to be protruding from the tail of the pancreas. Furthermore, these masses revealed ring-enhanced features, and the inner part showed no contrast effect, as if it were a necrotic tissue. The boundary between the tumor and the left diaphragm was unclear, suggesting invasion of the tumor. Although no dilatation of the main pancreatic duct or swelling the surrounding lymph node was observed, the larger tumor was enlarged compared with 1 month before (Fig. 1b), and had not been recognized 6 months previously (Fig. 1c). However, the diameter of the smaller tumor had not changed for 3 years (Fig. 1a–d). These masses showed low signal intensity on T1-weighted MRI (Fig. 2a), heterogeneous high intensity on T2-weighted MRI (Fig. 2b), and high signal intensity in diffusion-weighted imaging (DWI) (Fig. 2c), and the apparent diffusion coefficient (ADC) value revealed low signal intensity (Fig. 2d). Whole-body FDG-PET showed abnormal uptake in the two tumors (Fig. 3), and the standardized uptake value maximum was 13.1, with the delayed scan showing 17.4. No other abnormal uptake suggestive of distant metastasis, lymph node metastasis, or dissemination was observed. Examinations of the pancreatic juice cytology, EUS, and endoscopic retrograde cholangiopancreatography were difficult to perform because the patient had undergone total gastrectomy and pancreatoduodenectomy with gastrointestinal reconstruction. Finally, these tumors were diagnosed as suspected anaplastic pancreatic cancer or benign cystic tumor. In this case, during two previous surgeries for advanced gastric cancer and carcinoma of the ampulla of Vater, regional lymph node dissection was performed. In addition, because the tumors were located at the end of the pancreas tail, a sufficient surgical margin was considered to be easily obtained. On the basis of these findings, although there was a possibility of malignant disease, we conducted a partial resection of the remnant pancreas with concomitant resection of the left diaphragm as a diagnostic therapy to preserve pancreatic functions. In fact, intraoperative ultrasound examination revealed that a surgical margin of 20 mm from the tumor to the stump could be obtained, and histopathological examination of intraoperative frozen sections showed negative findings at the pancreatic stump. Macroscopically, the cystic lesion was 9 mm in diameter and was filled with white-yellowish material in the pancreatic tissue (Fig. 4). The other mass was 23 mm in diameter and presented intervening pancreatic tissue. In the former, histopathological examination revealed squamous epithelium on a background of cysts with proliferated lymphoid tissue (Fig. 5a). In the latter, neutrophil infiltration, lymphocytes, and collagen fibers were observed; thus, it was suggested that there was acute exacerbation of chronic inflammation. The histopathological diagnosis was an LEC with chronic abscess in the pancreas. Although tight adhesion was intraoperatively observed between the tumors of the pancreatic tail and the left diaphragm, which was suspected to be invasion of cancer to the diaphragm, histopathological examination demonstrated that the tumors were LEC with an adjacent abscess with tight adhesion to the left diaphragm.

The patient was discharged on postoperative day 10 without any complications. There were no signs of recurrence on follow-up imaging examinations performed 1 year after surgery.

## Discussion

LEC is defined as a rare benign disease that is characterized by a squamous-lined cyst of the pancreas that is filled with keratinous material and surrounded by dense lymphoid tissue [6, 7]. Lüchtrath et al. [8] first described this disease in 1985, and Truong et al. [9] named the cyst as “LEC of the pancreas”. LEC tends to arise in middle-aged to elderly men [10]. Almost all patients with LEC are asymptomatic, and physical examination is usually unremarkable. Serum levels of CA 19-9 are often elevated [11, 12]. CT examination often shows a well-defined solid cystic lesion and a ring-enhanced thick wall. MRI shows low signal intensity on T1-weighted MRI, high intensity on T2-weighted MRI, and high signal intensity in DWI [13]. In the present case, two cysts increased in size under follow-up, and this part of the tumor was visualized on PET as abnormal uptake of FDG, especially because the adjacent abscess made the diagnosis difficult.

Although many studies have been reported, it remains extremely difficult to diagnose LEC correctly and to rule out malignancies based on the preoperative imaging examinations. It is important to differentiate LEC from malignant pancreatic tumors to avoid unnecessary surgery [14]. In general, measurement of tumor markers may be useful in differentiating malignancies originating from benign tumors. In fact, the serum concentrations of tumor markers including CA 19-9 were within normal limits in the present case. However, several previous studies reported that serum CA 19-9 levels were elevated in more than 50% of cases with LEC [6, 15]. These findings suggest that elevated serum CA19-9 levels may lead to the false diagnosis of a malignant tumor in patients with LEC.

Recently, EUS-FNA biopsy was reported to facilitate the accurate diagnosis of LEC in 21% of cases [15]. Davakis et al. [16] reported that EUS is useful for diagnosing LEC because it may reveal cystic lesions on the pancreas, indicating a potential LEC. In addition, cytological examination via EUS-FNA may set the diagnosis. However, in some cases, previously performed abdominal surgery with gastrointestinal reconstruction may not allow for the examination procedure, such as in the present case. Furthermore, the indication of biopsy for potential malignant tumors remains controversial because of the risk of tumor dissemination [3].

Although FDG-PET has been reported to play an important role in the differential diagnosis of malignant or benign tumors, there are surprisingly few reports describing the usefulness of FDG-PET in the diagnosis of LEC. We searched PubMed using the keyword “pancreatic lymphoepithelial cyst’’ for the period of 1987–2021. The clinical features of patients who underwent FDG-PET to diagnose LEC in previous reports are summarized in Table 1 [4, 5, 17–19]. Almost all cases were middle-aged, and only one was female. Three cases showed abnormal uptake including our case, and two of three cases revealed non-inflammation of the pancreatic background. EUS-FNA was performed in two cases, and elevated serum levels of CA 19-9 were observed in only one case. However, the SUV of the tumors in the present case was higher than that in the previously reported cases. In the present case, abnormal FDG uptake was demonstrated continuously in the adjacent two tumors between LEC and the abscess. Furthermore, histopathological examination revealed that neutrophilic infiltration continued from the abscess to LEC. These findings suggest that high FDG uptake in the present case might be associated with the abscess rather than LEC itself. These results highlight the difficulty of accurate preoperative diagnosis of LEC, even using FDG-PET. Furthermore, one reason that made the diagnosis difficult in this case was the formation of the adjacent abscess. The patient had no other specific medical history, including pancreatitis, postoperative pancreatic fistula, and abdominal abscess. In addition, there was no sign of blood examination or symptom suggesting inflammation during follow-up. However, similarities between components in the abscess and LEC could not be revealed by histopathological examination. On the basis of these findings, although rupture of LEC was considered as a cause of the adjacent abscess formation, a definitive diagnosis was difficult.

**Table 1:** Previous case reports of lymphoepithelial cyst in whom FDG-PET was performed

No.	Author	Year	Age	Sex	Serum CA19-9	FDG-PET	EUS-FNA	Preoperative diagnosis	Surgical procedures	Texture of pancreas
					(U/ml)					
1	Maekawa H [17]	2009	58	Male	N/A	Positive	No	Pancreatic cystic neoplasm	Distal pancreatectomy	Normal
3	Yanagimoto H [18]	2013	53	Male	N/A	Negative	Yes	NET, MCN, IPMN	Distal pancreatectomy and splenectomy	Normal
2	Sasaki S [4]	2014	54	Male	28.9	Negative	No	LEC, MCN, pseudocyst	Enucleation	Normal
4	Satoh D [5]	2015	63	Male	85.5	Positive	Yes	Pancreatic cancer	Distal pancreatectomy and splenectomy	Normal
5	Ryu HD [19]	2015	44	Female	3.9	Negative	No	Pancreatic cancer	Distal pancreatectomy and splenectomy	Normal
6	Present case	2021	65	Male	6.1	Positive	No	Pancreatic cancer	Partial pancreatectomy	Chronic abscess

*FDG-PET* fluorodeoxyglucose-positron emission tomography

*EUS-FNA* endoscopic ultrasound guided fine-needle biopsy

*N/A* not applicable

*NET* neuroendocrine tumor

*MCN* mucinous cystic neoplasm

*IPMN* intraductal papillary mucinous neoplasm

*LEC* lymphoepithelial cyst

## Conclusions

We present a rare case of LEC with an abscess for which preoperative diagnosis was difficult even with FDG-PET. An experimental modality that can diagnose LEC accurately before surgery is yet to be established; therefore, surgical resection is considered unavoidable in cases in which malignancies cannot be ruled out through preoperative examinations.

## Data Availability

All data generated during this study are included in this published article.

## Abbreviations

LEC: Lymphoepithelial cyst
EUS-FNA: Endoscopic ultrasound guided fine-needle biopsy
CT: Computed tomography
MRI: Magnetic resonance imaging
FDG-PET: Fluorodeoxyglucose-positron emission tomography
CA19-9: Carbohydrate antigen 19-9
DWI: Diffusion-weighted imaging
